# Optimization of microwave-assisted extraction of zerumbone from *Zingiber zerumbet* L. rhizome and evaluation of antiproliferative activity of optimized extracts

**DOI:** 10.1186/s13065-016-0235-3

**Published:** 2017-01-05

**Authors:** Ali Ghasemzadeh, Hawa Z. E. Jaafar, Asmah Rahmat, Mallappa Kumara Swamy

**Affiliations:** 1 Department of Crop Science, Faculty of Agriculture, Universiti Putra Malaysia, 43400 Serdang, Selangor Malaysia; 2 Department of Nutrition & Dietetics, Faculty of Medicine & Health Sciences, Universiti Putra Malaysia, 43400 Serdang, Selangor Malaysia

**Keywords:** Response surface methodology, Zerumbone, Antiproliferative activity, HeLa cancer, Microwave extraction

## Abstract

**Background:**

The extraction of bioactive compounds from herbal materials requires optimization in order to recover the highest active dose. Response surface methodology was used to optimize variables affecting the microwave extraction of zerumbone from *Zingiber zerumbet* using the Box–Behnken design. The influence of variables, such as ethanol concentration (X_1_), microwave power (X_2_), irradiation time (X_3_), and liquid-to-solid ratio (X_4_), on the extraction of zerumbone was modeled using a second-order regression equation. The antiproliferative activity of optimized and non-optimized extracts was evaluated against the HeLa cancer cell line using the MTT (3-(4,5-dimethylthiazol-2-yl)-2,5-diphenyltetrazolium bromide) assay.

**Results:**

Two linear parameters, X_1_ and X_4_, and their quadratic parameters were highly significant at the P < 0.01 level. Two interaction parameters, X_1_X_4_ and X_2_X_3_ were significant, whereas interactions of X_1_X_2_, X_1_X_3_, X_2_X_4_ and X_3_X_4_ were insignificant (P > 0.05). The optimum microwave extraction conditions were as follows: ethanol concentration, 44%; microwave power, 518 W; irradiation time, 38.5 s; and liquid-to-solid ratio, 38 mL/g. Under these conditions, the maximum zerumbone yield was 5.88 mg/g DM, which was similar to the predicted value (5.946 mg/g DM). Optimized and non-optimized *Z. zerumbet* rhizome extracts exhibited significant antiproliferative activity against HeLa cancer cells, with half-maximal inhibitory concentration (IC_50_) values of 4.3 and 7.8 μg/mL, respectively, compared with 1.68 μg/mL for the anticancer drug cisplatin. When the extract concentration increased from 4.3 to 16.0 μg/mL, the inhibition of cancer cell growth increased from 50.0 to 79.5%.

**Conclusions:**

In this study, the optimized microwave protocol developed for extracting zerumbone from *Z. zerumbet* was faster and consumed less solvent than previous methods, while improving and enhancing the antiproliferative activity.

## Background

Herbs and spices produce a large number of phytochemicals, and have been used as food preservatives, flavorings, and traditional medicines for thousands of years. To date, a large number of studies in the medical industry have appraised phytochemicals to identify any biological activities with potential benefits for human health. The Zingiberaceae family of plants produces various compounds that are useful in the food industry, as seasonings and flavoring agents, and the pharmaceutical industry, as antimicrobial and antioxidant agents [1]. *Zingiber zerumbet* (L.) Roscoe ex Sm., known locally in Malaysia as “lempuyang,” or as “shampoo ginger” in English, is a plant that belongs to the Zingiberaceae family. *Z. zerumbet* is a perennial, tuberous root plant that grows naturally in damp and shaded parts of lowlands or hill slopes as scattered plants or thickets, and is native to India and the Malaysian Peninsula. *Z. zerumbet* is a traditional folk remedy and reportedly possesses various phytochemicals and secondary metabolites with antitumor [2], antioxidant [3], antipyretic, analgesic, [4], antibacterial [5], anti-inflammatory, antiallergic [6], and antihypersensitive [7] activities. Extracts of *Z. zerumbet* rhizomes have been reported to contain zerumbone, humulene, and camphene [8]. The main component of the essential oil of *Z. zerumbet* herb is zerumbone ((2*E*,6*E*,10*E*)-2,6,9,9-tetramethylcycloundeca-2,6,10-trien-1-one), a monocyclic sesquiterpene containing a cross-conjugated dienone moiety [9]. Zerumbone has a wide range of pharmaceutical activity, such as antioxidant activity [10], anti-inflammation activity [11, 12] anticancer activity [13, 14], antibacterial and antimutagenic activity [15], antimicrobial activity [16] and antidiabetic activity [17]. With a lot of medicinal benefit of zerumbone it seems that optimization of extraction process of this compound from *Zingiber zerumbet* L. (the only natural source of zerumbone) is necessary.

In industrial process control, response surface methodology (RSM) is usually used to determine the levels of input variables that optimize a particular response [18–20]. RSM is a technique that consists of: (a) designing experiments to deliver adequate and reliable response measurements, (b) developing a mathematical model with the best fit to the data obtained from the experimental design, and (c) determining the optimal value of the independent variables that produce the maximum or minimum values of the response. From an industrial perspective, mathematical models of the microwave extraction of bioactive compounds from plant material are required to optimize and predict the process in order for it to replace conventional extraction methods. Appropriate and optimized processing conditions, such as extraction, are required for efficient recovery and cost effectiveness when used on an industrial scale. The aim of this study is to consider the effect of different extraction methods and solvents on the zerumbone content extracted from *Z. zerumbet* rhizome, and optimize the selected extraction method using response surface methodology. The antiproliferative activities of the optimized extracts against the HeLa cancer cell line will also be considered.

## Methods

### Plant sampling

Rhizomes of *Z. zerumbet* were collected from the Universiti Putra Malaysia (UPM) glasshouse complex, where they had been grown using a plantation method described in our previous report [1]. Samples were submitted to the Institute of Bioscience, UPM, and were identified as *Z. zerumbet* and voucher specimens were deposited at the herbarium of the Institute of Bioscience, Universiti Putra Malaysia.

### Extraction

#### Microwave extraction

Briefly, 10 mL of the selected solvents (ethanol, methanol, *n*-hexane and chloroform) was added to *Z. zerumbet* powder (2.0 g) in a 50-mL double-necked flask attached to a cooling system. The resultant mixture then underwent microwave extraction, after which the flask was removed and the solution was water-cooled to room temperature. The mixture was vacuum filtered through Whatman No. 1 filter paper and collected in a volumetric flask. The residue was freeze-dried and stored at −20 °C for future analysis.

#### Reflux extraction

Lyophilized rhizome samples (2.0 g) were extracted with the 10 mL of selected solvents (ethanol, methanol, *n*-hexane and chloroform). Extraction was performed by refluxing at 65 °C for 35 min, after which the solution was cooled to room temperature and filtered through Whatman No. 1 filter. paper. Excess solvent was removed using a rotary evaporator and the resultant residue was freeze-dried and stored at −2.0 °C for future analysis.

#### Sonication

Lyophilized rhizome samples (2.0 g) were extracted with the 10 mL of selected solvent (ethanol, methanol, *n*-hexane and chloroform). The resultant mixture was sonicated at 65 °C for 35 min, after which the solution was cooled at room temperature and filtered through Whatman No. 1 filter paper. Excess solvent was removed using a rotary evaporator and the resultant residue was freeze-dried and stored at −2.0 °C for future analysis.

### Isolation and identification of zerumbone

The chromatographic isolation of zerumbone was achieved using ultra-high performance liquid chromatography (UHPLC; Agilent, Model 1200) with an Agilent C18 reversed-phase column (5 μm particle size, 4.6 × 150 mm). A gradient elution with a mobile phase comprising 0.1% formic acid in water (A) and methanol (B) was used as follows: 0 min, 60% B; 16 min, 60% B; 16.1 min, 100% B; 20 min, 100% B; 20.1 min, 60% B; and 25 min, 60% B. The detector wavelengths were set at 270 nm. The flow rate and injection volume were 1.2 mL/min and 10 μL, respectively. The column temperature was set at 40 °C. Zerumbone was identified by comparing retention time of extract with retention time of standard, UV spectra, and UV absorbance ratios after co-injection of samples and standards. Zerumbone standard (CAS Number 471-05-6, ≥98%) was purchased from Sigma-Aldrich (M) Sdn Bhd, Selangor, Malaysia. Zerumbone was dissolved in methanol HPLC grade to make different concentration (100, 200, 400, 800 and 1600 µg/mL). Linear regression equations were calculated using Y = aX ± b, where X is the concentration of the zerumbone and Y the peak area of the zerumbone obtained from UHPLC. The linearity was established by the coefficient of determination (R^2^).

### Optimization of zerumbone extraction process

*Zingiber zerumbet* powder (2.0 g) was stirred in aqueous ethanol in preparation for extraction using the microwave extraction system. Range of variables were chosen based on higher content of zerumbone from the previous preliminary experiment (single factor method). The microwave extraction parameters were microwave power (400–600 W), extraction time (30–90 s), liquid-to-solid ratio (20–40 mL/g), and ethanol proportion (20–60%). Each extraction process was carried out in triplicate. After extraction, mixtures were vacuum filtered through Whatman No. 1 filter paper and collected in a volumetric flask. The residue was then freeze-dried and stored at −20 °C for future analysis.

### Experimental design and statistical analyses

RSM was conducted to determine the microwave optimized extraction process variables for the maximum recovery of zerumbone using a Box–Behnken design (Minitab, version 8.0.7.1, USA). Three replicates were used to evaluate the pure error. Experimental design software (DOE, Minitab) was used for regression analysis of the data to fit a second-order polynomial equation (quadratic model), according to the following general equation which was then used to predict optimum conditions for the extraction process.

$$Y = B_{0} + \sum\limits_{i = 1k}^{k} {B_{i} X_{i} } + \sum\limits_{I = 1k}^{k} {B_{ii} X^{2} + \sum\limits_{i > jk}^{k} {B_{ij} X_{i} X_{j} + E} }$$

where *Y* is the response function (zerumbone yield in this case); *B*_*0*_ is a constant coefficient; *Bi*, *Bii*, and *Bij* are the coefficients of the linear, quadratic, and interactive terms, respectively; and *Xi* and *Xj* are the coded independent variables. The regression coefficients of individual linear, quadratic, and interaction terms were determined according to the analysis of variance (ANOVA). In order to visualize the relationship between the response and experimental levels of each factor, and to deduce the optimum conditions, the regression coefficients were used to generate 3-D surface plots and contour plots from the fitted polynomial equation. The factor levels were coded as −1 (low), 0 (central point or middle), and +1 (high), respectively. The variables were coded according to the following equation:

$$Xi = \frac{{X_{i} - X_{0} }}{\Delta X}$$

where *Xi* is the (dimensionless) coded value of the variable *X*_*i*_; *X*_*0*_ is the value of *X* at the center point, and Δ*X* is the step change.

### Determination of antiproliferative activity

#### Cell culture and treatment

HeLa cells and normal human mammary epithelial cells (MCF-10A) were cultured in RPMI 1640 Medium (Roswell Park Memorial Institute) containing 10% fetal bovine serum (FBS). The cells were incubated overnight at 37 °C in 5% CO_2_ to allow attachment to the culture plates.

#### MTT (3-(4,5-dimethylthiazol-2-yl)-2,5-diphenyltetrazolium bromide) assay

The MTT assay was conducted as follows: Cells were seeded in 96-well plates at a density of 1 × 10^4^ cells/well in 100 μL of RPMI medium. After 24 h, the medium was removed and the cells were incubated for 3 days with RPMI medium in the presence or absence of *Z. zerumbet* rhizome extracts at various concentrations. The extract concentrations used ranged from 1 to 16 μg/mL. After incubation, 20 μL of MTT reagent was added to each well. The plate was then incubated again in a CO_2_ incubator at 37 °C for 4 h. The reduced formazan products were quantified by measuring the absorbance at 570 nm by using an enzyme-linked immunosorbent assay (ELISA) reader. Each point represents the mean of triplicate experiments. Cell viability was determined using the following formula:

$${\text{Viability }}\left( \% \right) = \left( {{\text{sample optical density}}/{\text{control optical density}}} \right) \times 100$$

## Results and discussion

### Effect of different solvents and extraction methods on zerumbone content recovery

Many steps are involved in obtaining phytochemicals from plants, including milling, grinding, homogenization, and extraction. Among these steps, extraction has the greatest effect on the recovery and isolation of phytochemicals from plant material. Extraction efficiency is affected by the chemical nature of the phytochemicals, extraction method, sample particle size, extraction solvent, and the presence of interfering substances. Impact of different extraction methods (reflux, microwave, soxhlet and sonication) and solvents (ethanol, methanol, *n*-hexane and chloroform) on zerumbone content from the *Z. zerumbet* rhizomes was evaluated (Table 1). Significant differences was observed among different extraction methods and solvents. The lowest concentration of zerumbone (2.66 mg/g DM) was detected form chloroform solvent and soxhlet extraction method, while, the highest concentration (4.82 mg/g DM) was observed in ethanol solvent and microwave extraction method. Of all the solvent systems tested, ethanol proved to be the most efficient solvent for zerumbone extraction (Table 1). The highest zerumbone content was observed using ethanol solvent in microwave extraction, followed by sonication extraction. A similar result was reported previously in the microwave-assisted solvent extraction of effective constituents from *Herba epimedii* [21].

**Table 1:** Effect of different extraction solvents and methods on zerumbone content of *Z. zerumbet*

Extraction methods	Ethanol	Methanol	*n*-Hexane	Chloroform
Reflux	3.76 ± 0.03^c^	3.24 ± 0.08^f^	3.26 ± 0.07^f^	2.81 ± 0.01^i^
Microwave	4.82 ± 0.07^a^	4.06 ± 0.05^b^	4.07 ± 0.05^b^	3.36 ± 0.02^e^
Soxhlet	3.70 ± 0.06^c^	3.12 ± 0.02^h^	3.18 ± 0.04^g^	2.66 ± 0.01^j^
Sonication	4.11 ± 0.10^b^	3.55 ± 0.04^d^	3.25 ± 0.06^f^	3.1 ± 0.03^h^

All analyses are the mean of triplicate measurements ± standard deviation. Means not sharing a common letter were significantly different at P < 0.05. Unit is: mg/g DM

### Analysis of single factor method

#### Influence of ethanol concentration

The recovery of zerumbone from the *Z. zerumbet* rhizome with respect to ethanol concentration followed a parabolic curve from 20 to 60% ethanol (Table 2). The zerumbone content increased with increasing ethanol concentration in the extraction medium, up to 40%. A lower ethanol concentration in water can access the cells easily, but a high ethanol concentration can cause protein denaturation, preventing the dissolution of zerumbone and influencing the extraction rate. Ethanol is less polar while, water is strong polar solvent, and its suggested that they can blended together in any proportion [22]. It was provided that with decreasing of ethanol volume in ethanol/water solution the polarity of the solvent mixture will increase continuously [23]. Zerumbone is also a polar molecule, thus the zerumbone content in the extract increased with increasing water content, according to the “like dissolves like” principle [24]. In current study, when the ratio of ethanol/water decreased from 100 to 40%, the zerumbone recovery increased significantly from 3.25 to 4.87 mg/g DM. This might be attributed to the different dielectric properties of the solvents towards microwave heating, which play an important role in microwave extraction by facilitating heat distribution throughout the sample. In this study, 40% ethanol absorbed microwave energy relatively well, and was a good extraction solvent. The proportion of ethanol in the extraction solvent was examined at levels of 20–60% during optimization.

**Table 2:** Effect of single-factors on extraction of zerumbone from Z. zerumbet rhizome using microwave extraction method

Single-factor experiments							
Ethanol/water (% v/v)		Irradiation time (s)		MW Power (W)		Liquid-to-solid ratio (mL/g)	
(% v/v)	Zerumbone (mg/g DM)	(s)	Zerumbone (mg/g DM)	(W)	Zerumbone (mg/g DM)	Ratio (mL/g)	Zerumbone (mg/g DM)
20	4.11 ± 0.17^b^	30	4.15 ± 0.10^d^	300	3.78 ± 0.09^c^	10	3.26 ± 0.08^e^
40	4.87 ± 0.13^a^	60	5.1 ± 0.18^a^	400	4.32 ± 0.12^b^	20	3.88 ± 0.09^d^
60	3.66 ± 0.08^c^	90	4.86 ± 0.15^b^	500	4.65 ± 0.10^a^	25	4.21 ± 0.16^c^
80	3.43 ± 0.14^d^	120	4.45 ± 0.16^c^	600	3.55 ± 0.08^d^	30	5.08 ± 0.18^a^
100	3.25 ± 0.09^e^	180	4.30 ± 0.17^c^	700	3.21 ± 0.08^e^	40	4.76 ± 0.15^b^
		210	3.52 ± 0.11^e^	800	2.91 ± 0.06^f^		

All analyses are the mean of triplicate measurements ± standard deviation. Means not sharing a common letter in each column were significantly different at P < 0.05

#### Influence of microwave power

The effect of microwave power on the zerumbone recovered from *Z. zerumbet* rhizome was investigated in the range 300–800 W using a fixed solvent concentration (40% ethanol), an irradiation time of 2 min, and a liquid-to-solid ratio of 20 mL/g. A significant increase in the zerumbone recovery, from 4.32 to 4.65 mg/g DM, was observed at power levels of 400–500 W (Table 2). However, the recovery reduced significantly beyond 600 W, with the lowest recovery observed at 800 W (2.91 mg/g DM). The lower zerumbone recoveries above 600 W might be due to thermal degradation of the phytochemicals at higher microwave power levels. The heat generated by microwaves, with volumetric heating, in the plant cells could be strong enough to breakdown the phytochemicals, resulting in them not being recovered at higher power levels. These observations demonstrated that extractions at higher microwave output power levels, usually more than 800 W, did not ensure a better recovery of zerumbone than extractions at medium power. Based on our observations, microwave power levels of 400, 500, and 600 W were selected as the lower, middle, and upper levels, respectively, applied in RSM optimization.

#### Influence of irradiation time

In this study, zerumbone recovery was examined at different irradiation times (30–210 s) with three other fixed variables: solvent, 40% ethanol; microwave power level, 500 W; and liquid-to-solid ratio, 20 mL/g (Table 2). The results indicated that the zerumbone recovery increased with increasing microwave irradiation time at the beginning of extraction, with a maximum of 5.1 mg/g DM achieved in 60 s, followed by a decrease after 90 s. According to these results, an irradiation time of 30–90 s was used for further microwave extractions for RSM optimization.

#### Influence of extraction liquid-to-solid ratio

As in other extraction techniques, the liquid-to-solid ratio is an important parameter influencing zerumbone recovery. For example, in an industrial extraction process, it is important to maximize the extraction yield and minimize solvent consumption [25]. In the current study, the zerumbone recovery from *Z. zerumbet* rhizome increased with increasing liquid-to-solid ratio during extraction (Table 2). The maximum zerumbone recovery (5.08 mg/g DM) was observed at a liquid-to-solid ratio of 30 mL/g. A ratio of 20–40 mL/g was used during further optimization of process parameters for microwave extraction.

### Optimization of microwave extraction conditions

#### Modeling and model fitting using RSM

The regression coefficients of the intercept, linear, quadratic and interaction terms of the model for optimization of zerumbone extraction were calculated using the least square technique and are given in Table 3. The regression coefficients of the intercept, linear, quadratic, and interaction terms of the model were calculated using the least square technique, and are given in Table 3. The two linear parameters, ethanol concentration (X_1_) and liquid-to-solid ratio (X_4_), and their quadratic parameters were shown to be highly significant, at the level of P < 0.01, whereas interactions of X_1_X_2_, X_1_X_3_, X_2_X_4_ and X_3_X_4_ were insignificant (P > 0.05). The interactions X_1_X_4_ and X_2_X_3_ were also significant (P < 0.01). Discounting the non-significant parameters (P > 0.05), the following final predictive equation was obtained:

**Table 3:** Analysis of variance for the experimental results of zerumbone content from Z. zerumbet rhizomes

Parameter	Estimated coefficient	Standard error	Degree of freedom	Sum of squares	F value	Prob > F
Model intercept B_0_	5.51	0.084	14	3.95	13.38	0.0001
Linear						
X_1_	0.14	0.042	1	0.25	11.74	0.005
X_2_	0.017	0.042	1	0.00343	0.16	0.693
X_3_	−0.055	0.042	1	0.037	1.77	0.211
X_4_	0.27	0.042	1	0.085	40.46	0.0001
Quadratic						
$X_{1}^{2}$	−0.41	0.063	1	0.89	42.02	0.0001
$X_{2}^{2}$	−0.096	0.063	1	0.05	2.35	0.1513
$X_{3}^{2}$	−0.085	0.063	1	0.039	1.84	0.2001
$X_{4}^{2}$	−0.39	0.063	1	0.081	38.52	0.0001
Interaction						
X_1_X_2_	0.12	0.073	1	0.057	2.71	0.1259
X_1_X_3_	0.14	0.073	1	0.079	3.74	0.077
X_1_X_4_	−0.31	0.073	1	0.039	18.45	0.001
X_2_X_3_	0.45	0.073	1	0.08	37.78	0.0001
X_2_X_4_	−0.13	0.073	1	0.072	3.42	0.0894
X_3_X_4_	−0.061	0.073	1	0.015	0.69	0.4212
Lack of fit			10	0.23	2.15	0.3596
Pure error			2	0.022		
Residual			12	0.025		
R^2^ adjusted					0.875	
R^2^					0.939	
C.V.%	2.86					
Corr. total			26	4.21		

X_1_, ethanol concentration; X_2_, microwave power; X_3_, irradiation time; X_4_, liquid-to-solid ratio

$$\begin{aligned} {\text{Y }}\left( {\text{zerumbone}} \right) &= + 5.51 + 0.14{\text{X}}_{1} + 0.27{\text{X}}_{4} \\ & \quad{-}\, 0.31{\text{X}}_{1} {\text{X}}_{4} \,{-} \,0.16{\text{X}}_{1} {\text{X}}_{3} \,{-}\, 0.41{\text{X}}_{1}^{2} \,{-}\, 0.39{\text{X}}_{4}^{2}\end{aligned}$$

The ANOVA for the experimental results given in Table 3 shows that the model is significant, with an F value of 13.38. There is only a 0.01% chance that a “model F value” this large could occur due to noise. The determination coefficient (R^2^) was 0.9390, which implied that the sample variations of 93.9% for the microwave extraction efficiency of zerumbone from *Z. zerumbet* rhizomes were attributed to independent variables, with only 6.1% of the total variations not explained by the model. However, a large value of R^2^ does not always indicate that the regression model is sound [26]. In a good statistical model, the adjusted R^2^ (R^2^ adj.) should be close to R^2^. As shown in Table 2, the R^2^ and R^2^ adj. values for the model were close together. The “lack of fit F value” of 2.15 implied that the lack of fit was not significant relative to pure error (P > 0.0.5), confirming the validity of the model. The coefficient of variation (CV) value of 2.86% and the adequate precision ratio of 16.84 suggested that the model was reliable. In general, the results indicated that the model could work well for the prediction of zerumbone extracted from *Z. zerumbet* rhizomes.

#### Response surfaces analysis

**Fig. 1:**
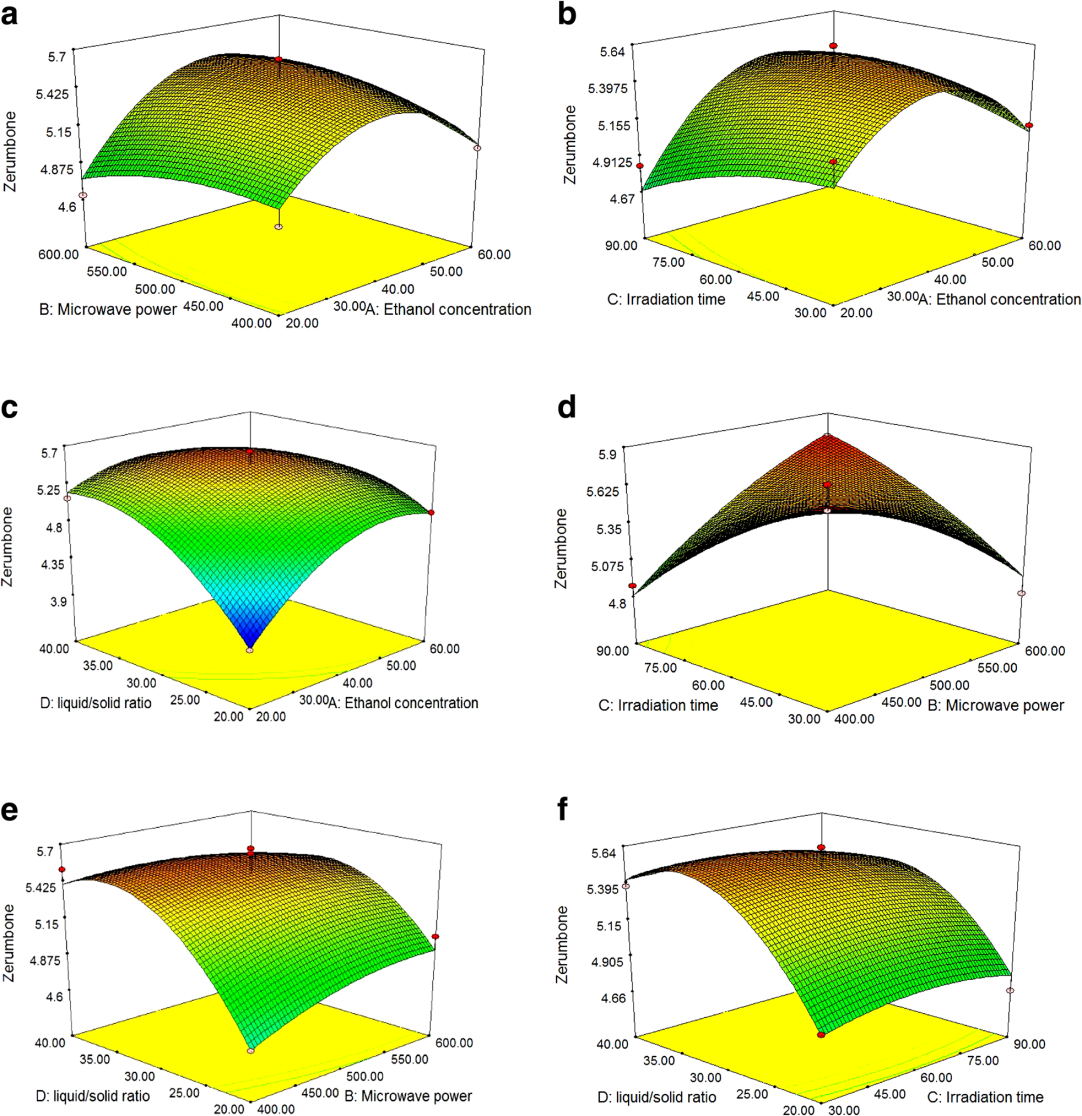
Response surface analysis for the zerumbone content from Z. zerumbet rhizome with microwave extraction with respect to ethanol concentration and microwave power (**a**); ethanol concentration and extraction/irradiation time (**b**); ethanol concentration and solvent-to-solid ratio (**c**); extraction/irradiation time and microwave power (**d**); microwave power and solvent-to-solid ratio (**e**); extraction/irradiation time and solvent-to-solid ratio (**f**)

To investigate the interactive effects of the independent variables and their mutual interaction on zerumbone extraction recovery, 3-D response surface profiles of multiple non-linear regression models were plotted (Fig. 1). The plots were generated by plotting the response on the *z*-axis against two independent variables, ethanol concentration (X_1_) and microwave power (X_2_), while keeping the other two independent variables [irradiation time (X_3_) and liquid-to-solid ratio (X_4_)] at their zero level.

Figure 1a depicts the relationship between ethanol concentration and each of the three other factors (MW power, irradiation time, and liquid-to-solid ratio) on zerumbone recovery. The zerumbone recovery from *Z. zerumbet* rhizome increased from 4.83 to 5.46 mg/g DW with increasing ethanol concentration (from 20 to 40%) and extraction power (from 400 to 500 W), nearly reaching a peak at 40% ethanol. Furthermore, the recovery was negatively affected by ethanol concentrations higher than 40% and additional extraction power. The best balance of ethanol concentration and extraction power should be sought to achieve the maximum zerumbone extraction rate. As shown in Table 3, the zerumbone recovery mainly depended on ethanol concentration, because its quadratic and linear effects were highly significant (P < 0.01), resulting in a curvilinear increase in zerumbone yield for all extraction powers and times tested (Fig. 1a, b).

The increase in zerumbone recovery suggested that zerumbone was more soluble in 45% ethanol/water, confirming the results of the single-factor experiments. Ethanol could facilitate an increase in extraction yield and water could enhance the swelling of cell material, favorably increasing the contact surface area between plant matrix and solvent, and resulting in an increased extraction yield [27]. Figure 1c shows the increase in zerumbone recovery with increasing ethanol concentration and extraction liquid-to-solid ratio at the beginning, followed by a decrease at medium values. The data suggested that the extraction liquid-to-solid ratio had a quadratic and linear effect (P < 0.01) on the zerumbone yield (Table 3). The zerumbone yield was maximized using a liquid-to-solid ratio of 30 over a range of the other operational factors (microwave power and irradiation time).

Increased microwave power with a longer irradiation time resulted in a continuous higher temperature in the extraction system. This combination of temperature and time was able to enhance the solubility of zerumbone and decrease the viscosity of the extraction solvent, thus accelerating the release and dissolution of zerumbone (Fig. 1d). Increasing the temperature could also aid the degradation of zerumbone to other compounds. The interaction effect of irradiation time (X_2_) and microwave power (X_3_) had a significant influence on the acquired ratio of zerumbone (P < 0.01). The linear and quadratic effects of these parameters (X_2_, X_3_) were insignificant (P > 0.05), while the synergistic effect was highly significant (P < 0.01). The zerumbone recovery using microwave energy was found to be a function of the interaction effect of extraction power and time.

In microwave extraction, microwave power was a key variable affecting the release of phytochemicals from different matrices by rupturing cell the wall, and also had the ability to modify equilibrium and mass transfer conditions during extraction. Increasing the microwave power accelerated zerumbone extraction. As shown in Fig. 1e, increasing the microwave power from 400 to 500 W resulted in a gradual increase in extraction yields of zerumbone, followed by a decline with a further increase in liquid-to-solid ratio (beyond 30 mL/g). This zerumbone yield trend could be attributed to the increase in liquid-to-solid ratio decelerating mass transfer, due to the lower heating efficiency under microwave conditions and zerumbone solubility. As shown in Fig. 1f, when the 3-D response surface plot was developed for zerumbone recovery with varying irradiation times and liquid-to-solid ratios, the best recovery of zerumbone was obtained using an extraction time of 60 s and liquid-to-solid ratio of 30 mL/g.

### Validation and verification of predictive model

The stationary point, denoting the maximum microwave extraction efficiency of zerumbone, was obtained using an experiment with the following critical values: ethanol concentration, 44%; microwave power, 518 W; irradiation time, 38.5 s; and liquid-to-solid ratio, 38 mL/g. The appropriateness of the model equation for predicting the optimum response values was tested using the above selected optimal conditions. The predicted extraction yield of zerumbone was 5.946 mg/g DM, which was consistent with the experimental yield of 5.88 mg/g DM. The predicted values were in close agreement with experimental values and were found to not be significantly different (P > 0.05) using a paired t test. The predicted response values deviated slightly from the experimental data.

### Antiproliferative activity

**Fig. 2:**
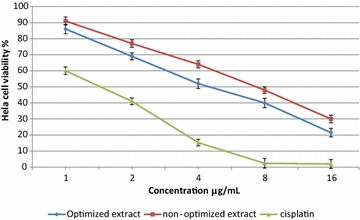
Antiproliferative activity of optimized and non-optimized extract of Z. zerumbet rhizome against HeLa cancer cell line. *Bars* represent standard errors of the means

**Fig. 3:**
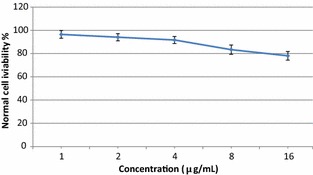
Cytotoxicity effect of optimized extract of Z. zerumbet rhizome against normal cell line. *Bars* represent standard errors of the means

Optimized extract (using RSM methodology) and non-optimized extract (primary extract, using ethanol as a solvent and microwave as a extraction method, Table 1) of *Z. zerumbet* rhizome were evaluated for their antiproliferative activity against the HeLa cancer cell line (Fig. 2). Significant differences in antiproliferative activity against the HeLa cancer cell line were observed between optimized and non-optimized extracts. Preliminary screening showed that the optimized and non-optimized extracts of *Z. zerumbet* rhizome exhibited significant antiproliferative activity against HeLa cancer cells, with half-maximal inhibitory concentration (IC_50_) values of 4.3 and 7.8 μg/mL, respectively. For comparison, the IC_50_ value for the anticancer drug cisplatin was 1.68 μg/mL. Lower IC_50_ values represent stronger cancer growth inhibition, at low concentrations. By optimizing the extraction process, the IC_50_ for HeLa cancer growth decreased from 7.8 to 4.3 μg/mL. For the optimized extract, increasing the concentration from 4.3 to 16 μg/mL increased the inhibition of cancer growth cells from 50 to 79.5%. Abdul et al. [28] showed that a purified zerumbone crystal from *Z. zerumbet* exhibited anticancer activity against the HeLa cancer cell line with an IC_50_ value of 2.50 μg/mL. In another study, zerumbone inhibited the growth of murine lymphoid tumor (P-388D) cells, induced DNA fragmentation in culture, and significantly prolonged the life of P-388D (1)-bearing CDF (1) mice (ILS% = 120.5) at a dosage of 2 mg/kg [29]. As shown in Fig. 3, normal cells treated with the optimized extract of *Z. zerumbet* rhizome at concentrations of 1–16 μg/mL showed a viability ranging from 96.4 to 78.1%. The antiproliferative activity of *Z. zerumbet* against a breast cancer cell line (MCF-7) has been reported, with an IC_50_ of 2.81 μg/mL [30]. Based on the Cytotoxicity Screening Index from the National Cancer Institute [31], IC_50_ value < 30 μg/mL is considered significant, while < 8 μg/mL is consider very significant. In this study, an IC_50_ value of 4.3 μg/mL was found for the optimized extract of *Z. zerumbet* rhizome, which was less than 8 μg/mL and considered very significant. In an herbal supplement, one ingredient may provide the desired therapeutic benefits while others may have toxic effects for humans. For example, many Malaysian herbs and spices might also contain toxic components that have been poorly investigated. According to the obtained results, optimized *Z. zerumbet* rhizome extracts showed nontoxic effects at the tested concentrations. Normal cells treated with *Z. zerumbet* extract, at concentrations ranging from 1 to 16 μg/mL, showed cell viabilities ranging from 96.5 to 78.1%. The antioxidant and anticancer activities of the herbal extracts are directly related to the phytochemicals or secondary metabolites of the extract [32–34].

## Conclusion

The results of this study indicated that zerumbone recovery of *Z. zerumbet* rhizome using microwave extraction was 17.9% higher than with sonication extraction, 28.1% higher than with reflux extraction, and 30.2% higher with than Soxhlet extraction. From an industrial perspective, the application of a microwave extraction method to the extraction of bioactive compounds from plant material requires a mathematical model to optimize and predict the process in order for it to replace conventional extraction methods. Appropriate and optimized processing conditions, such as extraction, are required for efficient recovery and cost effectiveness when used on an industrial scale. The present work established an improved and optimized procedure for extracting zerumbone from *Z. zerumbet* rhizome using a microwave extraction method. The following conditions were appropriate for the extraction of zerumbone from *Z. zerumbet* rhizome: Ethanol concentration, 44%; irradiation time, 38.5 s; microwave power, 518 W; and liquid-to solid ratio, 38 mL/g. The optimized extract showed significant antiproliferative activity against HeLa cancer cell lines without toxicity to normal cells.

## Abbreviations

DMSO: dimethyl sulfoxide
FBS: fetal bovine serum
IC_50_: half-maximal inhibitory concentration
MTT: (3-(4,5-dimethylthiazol-2-yl)-2,5-diphenyltetrazolium bromide)
UHPLC: ultra-high performance liquid chromatography
